# Bioinformatics classification of mutations in patients with Mucopolysaccharidosis IIIA

**DOI:** 10.1007/s11011-019-00465-6

**Published:** 2019-08-05

**Authors:** Himani Tanwar, D. Thirumal Kumar, C. George Priya Doss, Hatem Zayed

**Affiliations:** 1 Department of Integrative Biology, School of Bio Sciences and Technology, Vellore Institute of Technology, Vellore, Tamil Nadu 632014 India; 2 Department of Biomedical Sciences, College of Health and Sciences, Qatar University, Doha, Qatar

**Keywords:** Sanfilippo syndrome, Mucopolysaccharidosis IIIA, SGSH, nsSNPs, Molecular dynamics simulations analysis

## Abstract

Mucopolysaccharidosis (MPS) IIIA, also known as Sanfilippo syndrome type A, is a severe, progressive disease that affects the central nervous system (CNS). MPS IIIA is inherited in an autosomal recessive manner and is caused by a deficiency in the lysosomal enzyme sulfamidase, which is required for the degradation of heparan sulfate. The sulfamidase is produced by the N-sulphoglucosamine sulphohydrolase (*SGSH*) gene. In MPS IIIA patients, the excess of lysosomal storage of heparan sulfate often leads to mental retardation, hyperactive behavior, and connective tissue impairments, which occur due to various known missense mutations in the *SGSH*, leading to protein dysfunction. In this study, we focused on three mutations (R74C, S66W, and R245H) based on *in silico* pathogenic, conservation, and stability prediction tool studies. The three mutations were further subjected to molecular dynamic simulation (MDS) analysis using GROMACS simulation software to observe the structural changes they induced, and all the mutants exhibited maximum deviation patterns compared with the native protein. Conformational changes were observed in the mutants based on various geometrical parameters, such as conformational stability, fluctuation, and compactness, followed by hydrogen bonding, physicochemical properties, principal component analysis (PCA), and salt bridge analyses, which further validated the underlying cause of the protein instability. Additionally, secondary structure and surrounding amino acid analyses further confirmed the above results indicating the loss of protein function in the mutants compared with the native protein. The present results reveal the effects of three mutations on the enzymatic activity of sulfamidase, providing a molecular explanation for the cause of the disease. Thus, this study allows for a better understanding of the effect of SGSH mutations through the use of various computational approaches in terms of both structure and functions and provides a platform for the development of therapeutic drugs and potential disease treatments.

## Introduction

Mucopolysaccharidosis (MPS) IIIA, also known as Sanfilippo syndrome type A, is a neurodegenerative lysosomal storage disorder caused by a deficiency in the enzyme N-sulfoglucosamine sulfohydrolase (SGSH, EC:3.10.1.1), which is involved in the degradation of heparan sulfate. There are four different subtypes of MPS type III (type A – OMIM #252900, type B – OMIM #252920, type C – OMIM #252930, and type D – OMIM #252940) based on the enzyme deficiencies of SGSH, NAGLU, HGSNAT, and GNS, respectively. Each of the MPS III types is inherited in an autosomal recessive pattern with variations in the severity of phenotypes (Neufeld and Muenzer 1995). The genes encoding these four different enzymes have been characterized, and several mutations associated with these genes have been reported. The signs and symptoms of all four types are similar. Degeneration of the central nervous system, which results in mental retardation and hyperactivity, is the primary characteristic of MPS III, which commences in childhood (Fedele 2015). Other symptoms that are associated with the MPS III include delayed speech, behavioral problems, progressive dementia, macrocephaly, inguinal hernia, seizures, movement disorders, hearing loss, and sleep disturbances (Buhrman et al. 2014). The initial symptoms of the disease generally appear in the first to the sixth year of life, and death usually occurs in the early twenties (Valstar et al. 2010). The incidences of these subtypes are unevenly distributed. The estimated combined frequency of all four types varies between 0.28 and 4.1 per 100,000 live births. The incidence of MPS IIIA ranges from 0.68 per 100,000 to 1.21 per 100,000 in European countries (Baehner et al. 2005; Héron et al. 2011). MPS IIIA and MPS IIIB are more common than MPS IIIC and MPS IIID (Valstar et al. 2008), whereas MPS IIIA is more severe than MPS IIIB (Buhrman et al. 2013).

The gene encoding sulfamidase (*SGSH*), which was identified in 1995, is localized on chromosome 17q25.3. The 502 aminoacid sulfamidase protein contains five potential N-glycosylation sites (Scott et al. 1995). It spans 11 kb and contains eight exons (Karageorgos et al. 1996). Until now, 115 mutations, including missense/nonsense, deletions, insertions, and splicing, have been recorded for the SGSH protein according to the HGMD database (http://www.hgmd.cf.ac.uk/ac/all.php).

Proteins play a vital role in the regulation of various cellular functions, depending on their proper conformation in the cellular environment (Dill and MacCallum 2012). DNA variants known as single nucleotide polymorphisms (SNPs) have been known to introduce changes in the function of a gene (Cargill et al. 1999). A distinct class of such SNPs, known as nonsynonymous single nucleotide polymorphisms (nsSNPs), present in coding regions lead to amino acid changes that may cause alterations in protein function and account for vulnerability to disease. SNPs that do not affect the function of the protein are known as tolerated SNPs. Therefore, it is essential to distinguish the deleterious nsSNPs from the tolerant nsSNPs to understand the molecular genetic basis of human disease as well as to assess and understand the pathogenesis of the disease (Wang et al. 2009). Alterations and misfolding in protein structures due to nsSNPs lead to severe impairments that cause various diseases in humans (Chandrasekaran and Rajasekaran 2016; Thirumal Kumar et al. 2018a; Thirumal Kumar et al. 2018b; Valastyan and Lindquist 2014). Although most genetic variations in protein sequences are predicted to have very little or no effect on the function of the protein, some nsSNPs are known to be associated with the disease. These disease-related nsSNPs have adverse effects on the catalytic activity, stability, and interactions of the protein with other molecules. Thus, the identification of disease-associated nsSNPs is essential, and it will facilitate the elucidation of molecular mechanisms underlying a given disease (Sneha et al. 2017a; Zaki et al. 2017a). In subsequent years, the field of computational biology has emerged with advancements in automated methods to analyze the biological impact of nsSNPs based on the available information from modeled protein structures or structures derived from phylogenetic studies and comparative genomics (Chasman and Adams 2001; Sunyaev et al. 1999; Ng and Henikoff 2001). The experimental approach would be highly time-consuming to analyze the likely impact on protein function due to non-synonymous SNPs as well as to understand the association between these nsSNPs and the disease (Zhernakova et al. 2009). Information about the protein sequence and structure as well as the biochemical severity of the amino acid substitution, which are bioinformatics-based approaches, facilitates understanding of the phenotypic prediction. In recent years, various computational approaches have been developed that predict the effect of nsSNPs using various machine learning algorithms, such as the Hidden Markov model (Shihab et al. 2013), naïve Bayes classifier (Adzhubei et al. 2010), support vector machines (Acharya and Nagarajaram 2012; Capriotti et al. 2008), and neural network (Bromberg and Rost 2007), etc. In the present study, we performed an in silico analysis using various computational algorithms to explore the possible relationships between genetic mutations and phenotypic variations similar to our previous reports (Agrahari et al. 2018a; Agrahari et al. 2018b; Mosaeilhy et al. 2017a; Mosaeilhy et al. 2017b; Zaki et al. 2017b). To increase in prediction accuracy of disease causing variants, we used Meta-SNP server (Capriotti et al. 2013) that integrates four existing methods: PANTHER, SIFT, PhD-SNP, and SNAP to predict a mutation either disease (affecting the protein function) or neutral (having no impact). Further, a combination of these in silico tools and molecular dynamics studies in mutational analysis has been confirmed to be a dominant approach in understanding macromolecule behaviors and their microscopic interactions, allowing insights into the impact of mutations (Agrahari et al. 2019; Ali et al. 2017a; Ali et al. 2017b; Nagarajan et al. 2015; Sneha et al. 2018a; Sneha and George Priya Doss 2016; Sneha et al. 2018b; Thirumal Kumar et al. 2019). Molecular dynamics (MD) aid in understanding the significant changes in the macromolecular structures of proteins due to mutations at an atomic level. Various studies have been performed that show the influence of MDS in analyzing the effects of nsSNPs on protein structure (George Priya Doss and NagaSundaram 2012; Nagasundaram and George Priya Doss 2013; Thirumal Kumar et al. 2018a; Thirumal Kumar et al. 2018b; Xu et al. 2018; George Priya Doss and Zayed 2017; Mosaeilhy et al. 2017a, b; Sneha et al. 2017b; John et al. 2013).

Based on experimental studies (Esposito et al. 2000; Héron et al. 2011; Knottnerus et al. 2017; Muschol et al. 2004; Perkins et al. 1999; Sidhu et al. 2014; Trofimova et al. 2014; Weber et al. 1997), the missense mutations R74C, S66W, and R245H were subjected to prediction tools. The goal of this study was to understand the impact of these deleterious nsSNPs at the structural level. The models of the mutant proteins were generated based on the crystal structure of the SGSH protein. The native and mutant proteins were then subjected to MD simulation analysis using GROMACS to observe the structural changes. Therefore, the present study demonstrates the potential of using computational methods in resolving the effect of deleterious nsSNPs on protein structure.

## Materials and methods

### Datasets

The protein sequence of the SGSH protein in FASTA format was extracted from the UniProt database (UniProt ID: P51688) (http://www.uniprot.org/) (UniProt: A hub for protein information 2014). The PDB structure was retrieved from the Protein Data Bank (Berman et al. 2000) for structural analysis (PDB ID: 4MHX), and the literature study of the mutations associated with Sanfilippo syndrome was conducted using the OMIM (Online Mendelian Inheritance in Man) (Amberger et al. 2009) and NCBI PubMed databases.

### Prediction methods

In recent years, various in silico prediction methods have been developed to assess the effects of amino acid mutations on proteins and their function. Some prediction methods are based on the physicochemical properties of amino acids and the nature of their side chains, and some incorporate available annotations, e.g., Gene Ontology. There are classification methods, which are usually based on machine learning techniques such as neural networks, support vector machines, Bayesian methods, and mathematical operations. The computationally derived information about the structure and function of the protein and the properties of both the native and substituted amino acid residues are combined and finally characterize the mutation as disease-linked or neutral (Mueller et al. 2015).

### Pathogenic prediction of nsSNPs

The three mutants (R74C, S66W, and R245H) were subjected to computational prediction using Meta-SNP server (http://snps.biofold.org/meta-snp/index.html) (Capriotti et al. 2013). Meta-SNP computes the results based on the random forest binary classifier to discriminate between disease-related and polymorphic non-synonymous SNPs. This prediction tool comprises of other algorithm such as PANTHER (Mi et al. 2007), PhD-SNP (Capriotti et al. 2006), SIFT (Ng and Henikoff 2003), SNAP (Johnson et al. 2008), and Meta-SNP to predict the pathogenicity of the mutations. The scores range between 0 and 1, the score > 0.5 for the mutation is predicted to be disease.

- **PANTHER** (Protein Analysis Through Evolutionary Relationships)

PANTHER uses HMM-based statistical modeling methods and multiple sequence alignments to perform evolutionary analysis of coding nsSNPs. It estimates the likelihood of a particular nsSNP, causing a functional impact on the protein. The scores range between 0 and 1, the score > 0.5 for the mutation is predicted to be disease.

- **PhD-SNP** (Predictor of human Deleterious Single Nucleotide Polymorphisms)

PhD-SNP is based a SVM-based classifier. This is developed to predict the pathogenicity based on a single SVM trained and tested on protein sequence and profile information. The scores range between 0 and 1, the score > 0.5 for the mutation is predicted to be disease.

- **SIFT** (Sorting Intolerant From Tolerant)

SIFT classifies whether a mutation affect the protein function based on sequence homology and the physical properties of amino acids. This tool can be used to classify the naturally occurring mutations and laboratory-induced missense mutations. The values are in positive and the mutation score > 0.05 is predicted to be neutral.

- **SNAP** (Screening for Non-Acceptable Polymorphisms)

SNAP is a method based on neural networks that applies an advanced machine-learning approach to study the effects of nsSNPs. The prediction about the loss or gain of a protein’s function due to the amino acid substitution is depicted based on the information about the sequence and structural components, such as the secondary structure, solvent accessibility, and residue conservation within sequence families. The scores range between 0 and 1, the score > 0.5 for the mutation is predicted to be Disease.

### Stability prediction of nsSNPs

Prediction of protein stability changes resulting from single amino acid variations helps in understanding the structure of the protein. The stability analysis was performed using I-Mutant 3.0 (Capriotti et al. 2008), MUpro (Cheng et al. 2006), and SDM (Topham et al. 1997) to analyze the impact of deleterious variants on the SGSH protein.

- **I-Mutant 3.0**

I-Mutant 3.0 (http://gpcr2.biocomp.unibo.it/cgi/predictors/I-Mutant3.0/I-Mutant3.0.cgi) is a support vector machine (SVM)-based tool that automatically predicts protein stability changes upon single point mutations. This tool can be used as a classifier to predict the sign of the protein stability change following a mutation as well as a regression estimator to predict the deltaDeltaG values. The output file depicts the predicted free energy change (DDG), which is calculated from the unfolding Gibbs free energy change of the mutated protein minus the unfolding free energy value of the native protein (Kcal/mol). A value DDG > 0 shows increased stability, and DDG < 0 shows decreased stability (Capriotti et al. 2008).

- **MUpro**

The MUpro (http://mupro.proteomics.ics.uci.edu/) server based on two machine learning programs (SVM and Neural Networks) was used to predict protein stability changes for single amino acid mutations. The output of the program is the sign of the energy change (plus or minus). If the energy change ΔΔG value is positive, the mutation increases stability and is classified as neutral. If the ΔΔG value is negative, the mutation is destabilizing and classified as deleterious (Cheng et al. 2006).

- **SDM (**Site Directed Mutator)

SDM (http://mordred.bioc.cam.ac.uk/~sdm/sdm.php) is a statistical potential energy function that was developed by Topham et al. (Topham et al. 1997) to predict the effect of SNPs on the stability of proteins. It is a useful method for guiding the design of site-directed mutagenesis experiments or predicting the mutational impact on the protein structure. SDM calculates a stability score analogous to the free energy difference between the native and mutant proteins with the use of environment-specific amino acid substitution frequencies within homologous protein families. The method performs comparably or better than other published methods in classifying mutations as stabilizing or destabilizing (Worth et al. 2011).

### Mutation structural analysis

Based on experimental studies and the results obtained through computational analysis of nsSNPs using in silico tools, the three mutations were subjected to structural analysis. The mutations were induced based on the corresponding amino acid positions in the crystallized structure of the protein using the SWISS-PDB viewer (Guex and Peitsch 1997), and energy minimization was performed using the same software (Pettersen et al. 2004).

### Evolutionary conservation analysis

The ConSurf server (http://consurf.tau.ac.il) (Glaser et al. 2003) was used to calculate the conservation pattern of the SGSH protein to measure the degree of conservation at each aligned position. It first identifies conserved positions using multiple sequence alignment, then calculates the evolutionary conservation rate using empirical Bayesian inference and provides the evolutionary conservation profiles of structure or the sequence of the protein. The ConSurf score ranges from 1 to 9, with 1 representing rapidly evolving sites, 5 depicting the average, and 9 representing slowly evolving (evolutionary conserved) sites. Along with the conservation profile, the exposed and buried regions of the protein are also provided. This tool also predicts the structural/functional impact of the amino acid across the protein.

### Physicochemical property analysis

NCBI-Amino Acid Explorer (https://www.ncbi.nlm.nih.gov/Class/Structure/aa/aa_explorer.cgi) provides a detailed explanation of properties such as charge, size, hydrophobicity, hydrogen bonds, side-chain flexibility, etc., to evaluate the changes in the biophysical and chemical characteristics of the native and mutant amino acids (Bulka 2006).

### Salt bridge analysis

The energy-minimized structures of native and mutant proteins (R74C, S66W, and R245H) obtained from the Swiss-PDB viewer (Guex and Peitsch 1997) were used for the salt bridge prediction using the ESBRI (Costantini et al. 2008) web server. The server is based on a CGI script written in Perl language that finds existing interactions between oppositely charged groups and recognizes at least one Asp or Glu side-chain carboxyl oxygen atom and one side-chain nitrogen atom of Arg, Lys or His within a distance of 4.0 Å.

### Molecular dynamics

Molecular dynamics simulations were performed using the GROMACS package (Pronk et al. 2013) with the GROMOS96 43a1 force field (Schuler et al. 2001). The protein structure (PDB ID: 4MHX) was converted to a GROMACS file using pdb2gmx, and the hydrogen atoms were removed using the –ignh option. The models were centered in a cubical box of fixed volume filled with SPC/E water molecules and placed at least 1.0 nm from the edge of the box. The neutrality of the system was ensured by replacing the chlorine ions with sodium ions using genion. To escape steric clashes and an inappropriate geometry, energy minimization of the system was performed for 50,000 steps with a maximum force of 1000.0 KJ/mol/nm. To constrain the bond lengths, the steepest descent minimization algorithm was used. Electrostatic interactions were calculated using the Particle Mesh Ewald method (Darden et al. 1993; Essmann et al. 1995; Kholmurodov et al. 2000). Then, the equilibration process was carried out for the energy-minimized system using NVT (constant number of particles, volume, and temperature) and NPT (constant number of particles, pressure, and temperature) ensembles with the temperature maintained at 300 K and time constant at 1 ps using a Berendsen thermostat (Berendsen et al. 1984). This equilibrated system was then subjected to MDS for 30 ns, and trajectories such as g_rms, g_rmsf, g_hbond, g_gyrate, and g_sas utilities were used to analyze the results. The graphs were plotted using GRACE (Graphing, Advanced Computation, and Exploration).

### Secondary structure and surrounding amino acid changes between native and mutant proteins

To study the variations in secondary structure patterns, we performed a secondary structure analysis of the native and mutant proteins using the PDBsum database, which assigns various secondary structure labels to the residues of the protein (Laskowski et al. 1997). The secondary structural elements such as alpha helices, beta strands, beta sheets, beta bulges, strands, helices, helix-helix interactions, beta turns, and gamma turns were calculated for both the native and mutant solved structures of SGSH protein at the end of the 30ns simulation. Additionally, the residue changes within the 4 Å surroundings were also observed through PyMOL. The surrounding amino acid changes for the native and mutant proteins were identified from the point of the mutation.

### Principal component analysis (PCA)

PCA, also known as Essential Dynamics (ED), is a method that reduces the complexity of the data and explains the observed motional changes in the protein throughout the simulation (Amadei et al. 1993). For PCA, the rotational and translational movements were removed, and a variance/covariance matrix was constructed using the g_covar command. Next, the g_anaeig command was used to obtain the PCA of the protein, maintaining the covariance matrix as a starting point. The eigenvalues and eigenvectors, and their projection along with the first two principal components were calculated. A set of eigenvalues and eigenvectors was then identified by diagonalizing the matrix. The eigenvalue is a measure of distortion induced by the transformation, and eigenvectors elucidate this distortion. The trajectory files were analyzed, and the graph was plotted using the GRACE Program.

## Results

### Pathogenicity and stability predictions

The pathogenicity and stability predictions were made using different tools to predict the impact of mutations on the structure and function of the protein. The mutations R74C, S66W, and R245H were subjected to pathogenic prediction tools (Meta-SNP) and stability prediction tools (SDM, MUpro, I-Mutant 3.0). Mutations S66W and R74C were predicted to be “Disease” by all the prediction tools whereas, the mutation R245H was predicted to be “Neutral” by PhD-SNP and SIFT (Table 1). Considering the lower Reliability Index (RI) for R245H mutation, all the three mutations were further taken for stability analysis. From the stability analysis, all the three mutations were found to possess destabilizing effect. (Table 1).

**Table 1:** Pathogenicity and stability prediction

Mutation	S66W	R74C	R245H
PANTHER	Disease	Disease	Disease
Score^#^	0.938	0.985	0.666
PhD-SNP	Disease	Disease	Neutral
Score^#^	0.559	0.757	0.387
SIFT	Disease	Disease	Neutral
Score^#^	0	0	0.08
SNAP	Disease	Disease	Disease
Score^#^	0.75	0.865	0.67
Meta-SNP	Disease	Disease	Disease
Score^#^	0.814	0.902	0.627
RI*	6	8	3
SDM	Destabilizing	Destabilizing	Destabilizing
DDG^$^	−0.63	−1.82	−1.15
MuPro	Destabilizing	Destabilizing	Destabilizing
DDG^$^	−0.467	−0.584	−1.068
I-mutant 3.0	Destabilizing	Destabilizing	Destabilizing
DDG^$^	−0.34	−0.72	−1.71

*RI - Reliability Index between 0 and 10; # For PANTHER, PhD-SNP, SNAP, and Meta SNP tools: the score ranges between 0 and 1. If > 0.5 mutation is predicted Disease; for SIFT the score is Positive Value If > 0.05 mutation is predicted Neutral; $A value with DDG > 0 shows increased stability, and DDG < 0 shows decreased stability

### Conservation analysis

**Fig. 1:**
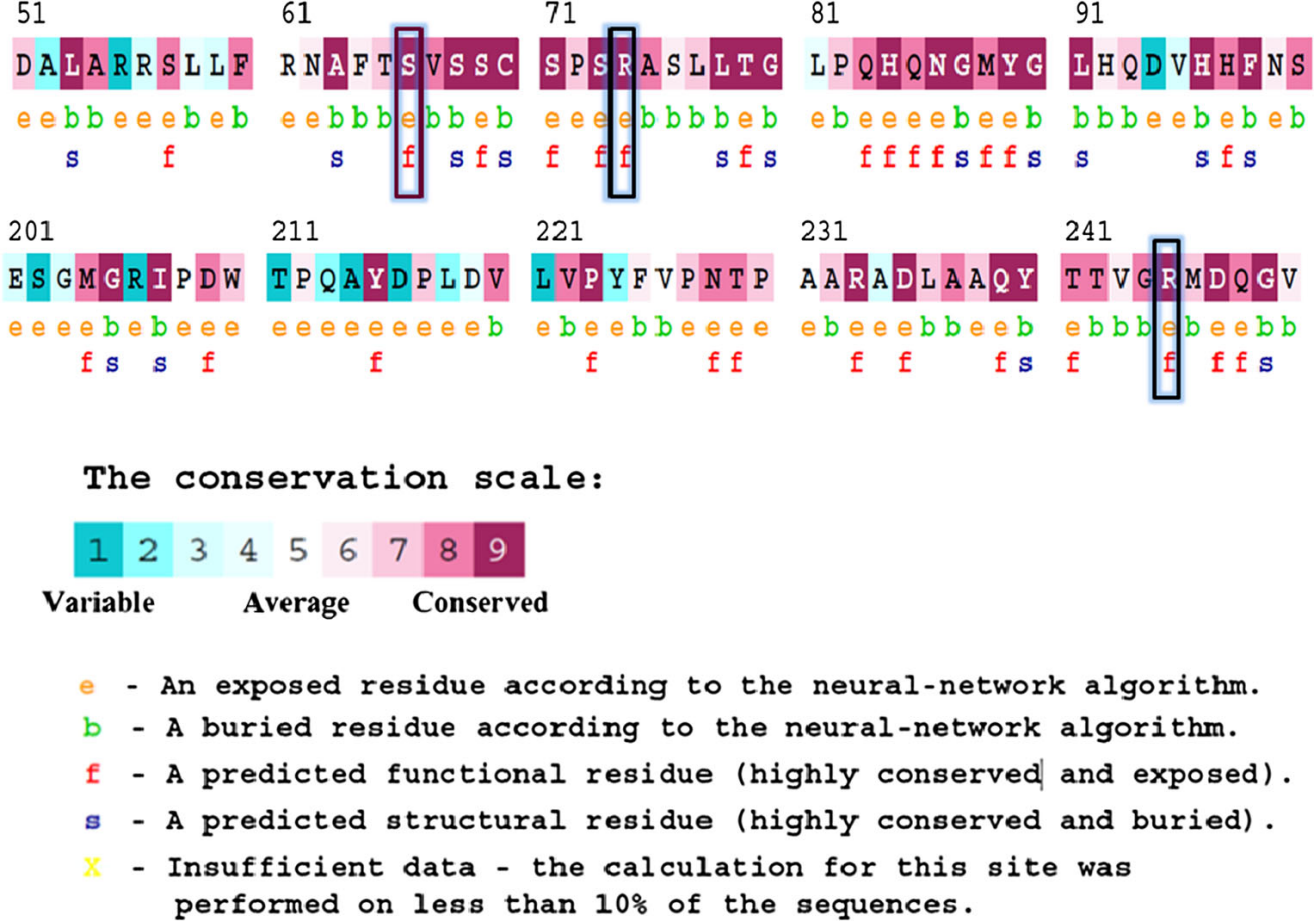
Conservation analysis of the protein sequence of SGSH using ConSurf. The positions R64, S66, and R245, are highly conserved with a score of 9 and present in exposed regions of the protein

The conservation pattern reveals the importance of a residue that helps to maintain the structure and function of a protein. ConSurf evaluates the degree of conservation at each aligned position, which represents the localized evolution (Glaser et al. 2003). It first identifies the conserved positions using Multiple Sequence Alignment and then measures the evolutionary conservation rate using an empirical Bayesian interface. The level of conservation of amino acids at positions R74, S66, and R245 were assessed using the ConSurf tool. A mutation in a more conserved position may affect the function of the protein. The results are shown in the figure, which shows that arginine at positions 74 and 245 as well as serine at position 66 displayed a conservation score of 9, thus predicting a highly conserved region across the species (Fig. 1). Therefore, mutations at positions R74, S66, and R245 might have deleterious effects on the protein. The solvent accessibility property of each amino acid was also assessed using the ConSurf results, which predicted all amino acid positions 74, 66, and 245 to be in exposed regions, which might have functional effects.

### Analysis of physicochemical properties

The physicochemical effects due to amino acid substitutions lead to local and global changes in the protein based on changes in size, charge, hydrophobicity, side-chain flexibility, hydrogen bonds, etc. These physicochemical properties were compared between the native and mutant proteins using the NCBI-Amino Acid Explorer tool (Table 2). The results demonstrate that the mutation of arginine to cysteine at position 74 resulted in an alteration of the side chain flexibility from high to low. The mode of interaction in arginine was found to consist of ionic and hydrogen bonds and van der Waals interactions, whereas cysteine contributed to covalent disulfide bonds and van der Waals interactions. There was a loss of hydrogen bonds, an increase in hydrophobicity, and a reduction in molecular weight. In the case of the mutation S66W, the side-chain flexibility was modified from low to moderate. The mode of interactions in serine consisted of hydrogen bonds and van der Waals interactions, whereas tryptophan resulted in hydrogen bonds, aromatic stacking, and van der Waals interactions. There was a loss of hydrogen bonds, an increase in hydrophobicity, and an increase in molecular weight. There was a change in polarity from polar to non-polar and aliphatic to aromatic properties. Mutation of arginine to histidine at position 245 resulted in the alteration from high to moderate side-chain flexibility. The interaction modes were ionic and hydrogen bonds and van der Waals interactions in both the native and mutant proteins, with the addition of aromatic stacking in the mutant protein. There was a decrease in hydrogen bonds, an increase in hydrophobicity, reduction in molecular weight, and conversion from aliphatic to aromatic properties (Table 2).

**Table 2:** Physicochemical properties of the native and mutants (R74C, S66W, and R245H) in the SGSH protein

Physicochemical Properties	R74C		S66W		R245H	
	Native (Arginine)	Mutant (Cysteine)	Native (Serine)	Mutant (Tryptophan)	Native (Arginine)	Mutant (Histidine)
Side-chain flexibility	High	Low	Low	Moderate	High	Moderate
Interaction mode	Ionic, H-bonds, van der Waals	Covalent disulfide bonds, van der Waals	H-bonds, van der Waals	H-bond, aromatic stacking, van der Waals	Ionic, H-bonds, van der Waals	Ionic, H-bonds, aromatic stacking, van der Waals
Potential side chain H-bonds	7	0	3	1	7	3
Residue Molecular Weight	156	103	87	186	156	137
Hydrophobicity	0.000	0.721	0.601	0.854	0.000	0.548
Polarity	Polar	Non-polar	Polar	Non-polar	Polar	Polar
General Property	Aliphatic	Aliphatic	Aliphatic	Aromatic	Aliphatic	Aromatic

### Salt bridge analysis

The number of salt bridges formed was calculated using the ESBRI online server by providing the atomic coordinates of the solved structures of the native and mutant proteins as input. Salt bridge formation is significantly influenced by the environment of the protein and depends on the ionization properties of the amino acids. The results indicated that 33 salt bridges were formed in the native and S66W mutant, whereas mutants R74C and R245H formed 30 and 32 salt bridges, respectively (Table 3).

**Table 3:** Number of salt bridges formation in the native and mutant proteins (R74C, S66W, and R245H)

Salt Bridges		Distance between residues of native and mutant proteins (Å)			
Residue 1	Residue 2	Native	R245H	R74C	S66W
ND1 HIS A 178	OD2 ASP A 31	2.91	2.89	2.89	2.90
ND1 HIS A 429	OD1 ASP A 426	2.84	2.82	2.82	2.82
ND1 HIS A 429	OD2 ASP A 426	3.92	3.94	3.94	3.95
ND1 HIS A 84	OD1 ASP A 477	3.98	3.99	3.99	3.99
ND1 HIS A 84	OD2 ASP A 477	3.76	3.77	3.78	3.78
NE2 HIS A 181	OD2 ASP A 32	3.96	3.93	3.93	3.93
NE2 HIS A 245	OD2 ASP A 179	–	3.56	–	–
NE2 HIS A 383	OD1 ASP A 440	–	3.99	3.99	4.00
NH1 ARG A 150	OD1 ASP A 179	2.62	2.72	2.74	2.71
NH1 ARG A 169	OD2 ASP A 135	3.98	–	–	–
NH1 ARG A 182	OD1 ASP A 235	3.01	3.03	3.03	3.03
NH1 ARG A 182	OD2 ASP A 235	3.27	3.23	3.22	3.24
NH1 ARG A 282	OD2 ASP A 32	3.08	3.07	3.07	3.08
NH1 ARG A 304	OE1 GLU A 355	3.62	3.58	3.58	3.58
NH2 ARG A 160	OE1 GLU A 256	3.80	3.82	3.82	3.82
NH2 ARG A 169	OD2 ASP A 167	3.28	3.27	3.27	3.27
NH2 ARG A 182	OD1 ASP A 235	3.80	3.87	3.87	3.84
NH2 ARG A 182	OD2 ASP A 235	2.85	2.89	2.89	2.88
NH2 ARG A 245	OD1 ASP A 179	2.98	–	2.97	2.97
NH2 ARG A 245	OD2 ASP A 179	3.77	–	3.83	3.81
NH2 ARG A 282	OD2 ASP A 399	2.59	2.75	2.75	2.71
NH2 ARG A 304	OE1 GLU A 355	2.75	2.76	2.76	2.75
NH2 ARG A 377	OD1 ASP A 477	2.90	2.94	2.94	2.93
NH2 ARG A 377	OD2 ASP A 477	3.27	3.37	3.37	3.35
NH2 ARG A 414	OD1 ASP A 410	3.93	3.93	3.93	3.93
NH2 ARG A 435	OD1 ASP A 484	3.41	3.36	3.36	3.38
NH2 ARG A 456	OD2 ASP A 454	2.73	2.76	2.76	2.75
NH2 ARG A 74	OD1 ASP A 31	2.93	2.97	–	2.96
NH2 ARG A 74	OD2 ASP A 273	3.14	3.12	–	3.12
NH2 ARG A 74	OD2 ASP A 31	3.86	3.92	–	3.91
NZ LYS A 123	OD1 ASP A 31	3.22	3.27	3.27	3.26
NZ LYS A 123	OD2 ASP A 31	2.86	2.91	2.91	2.90
NZ LYS A 124	OE1 GLU A 129	2.96	2.98	2.97	2.98
NZ LYS A 156	OD1 ASP A 209	3.60	3.61	3.61	3.60
NZ LYS A 156	OD2 ASP A 209	2.90	2.92	2.92	2.91
Total no. of salt bridges		33	32	30	33

### Protein structure conformational flexibility and stability analysis

**Fig. 2:**
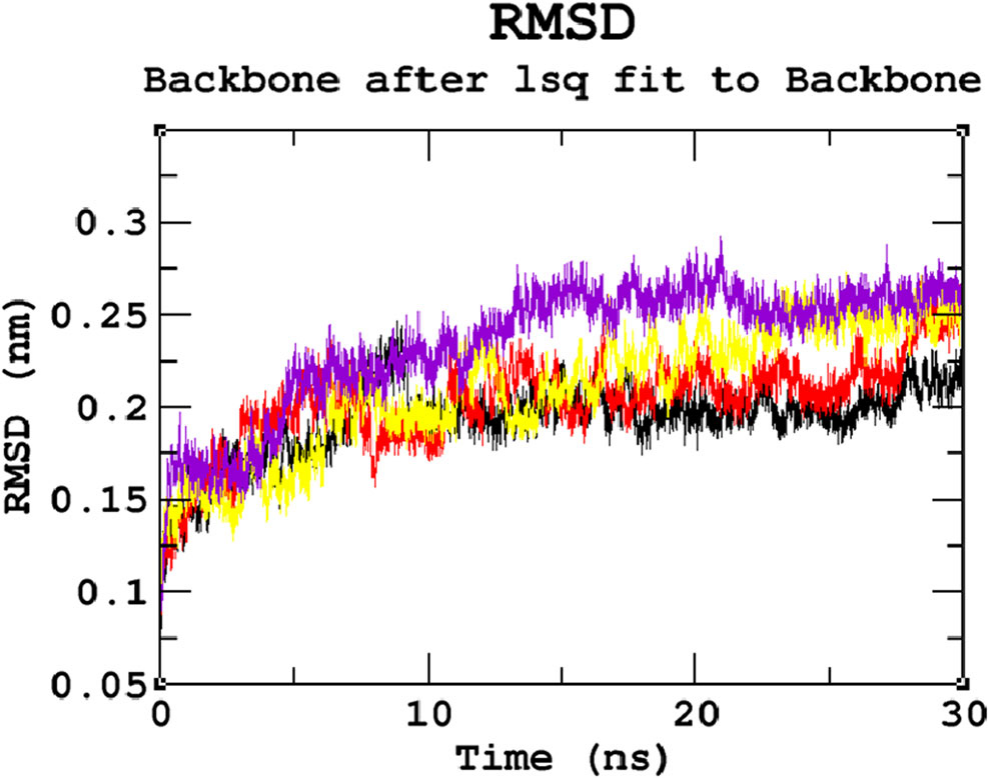
Backbone Root Mean Square Deviation (RMSD) graph for the 30ns MDS of native and mutant proteins. Color scheme: (**a**) native (black), (**b**) R74C (red), (**c**) S66W (yellow), and (**d**) R245H (violet)

**Fig. 3:**
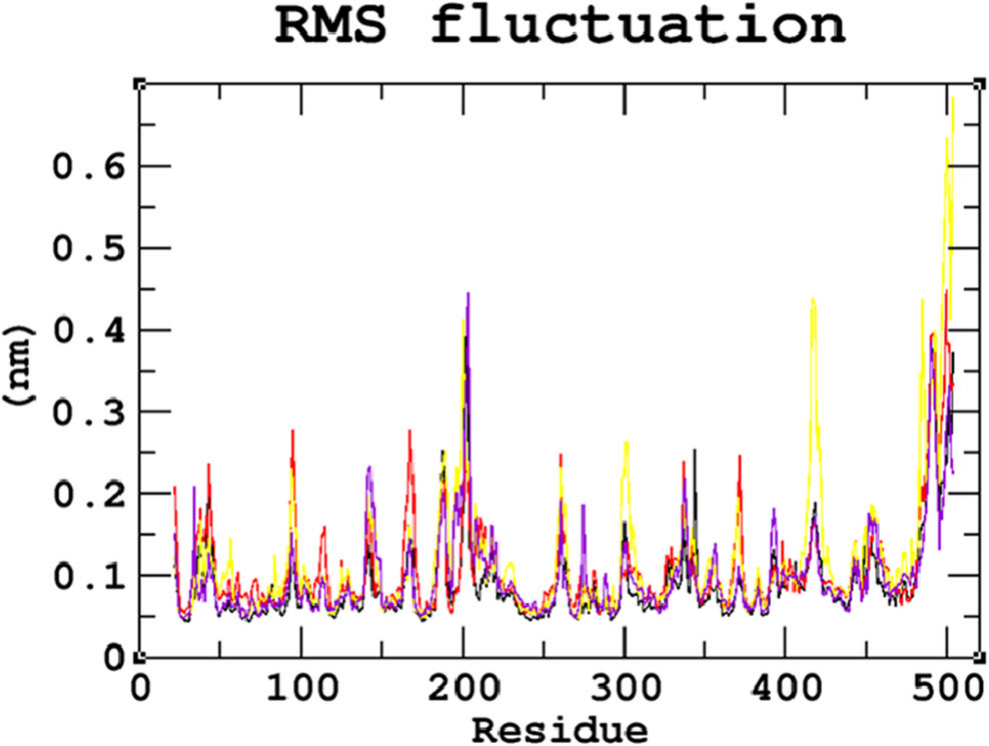
Root Mean Square Fluctuation (RMSF) graph for the 30ns MDS of native and mutant proteins. Color scheme: (**a**) native (black), (**b**) R74C (red), (**c**) S66W (yellow), and (**d**) R245H (violet)

**Fig. 4:**
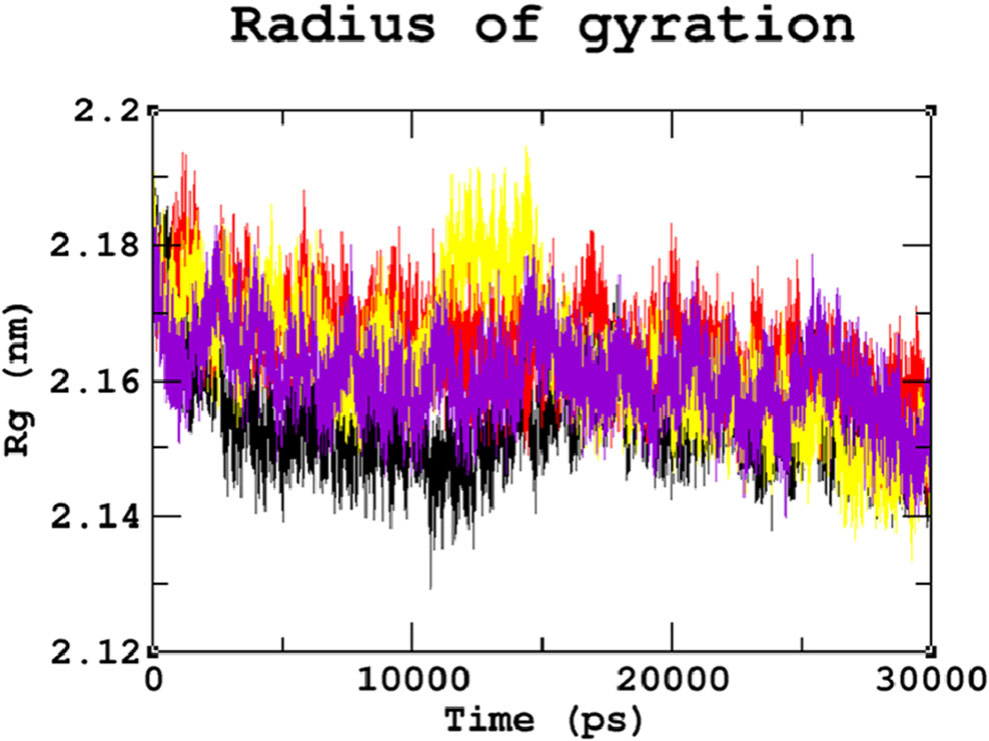
Radius of gyration (Rg) graph for the 30ns MDS of native and mutant proteins. Color scheme: (**a**) native (black), (**b**) R74C (red), (c) S66W (yellow), and (d) R245H (violet)

MDS studies were performed for 30 ns to analyse the atomic level changes in SGSH protein concerning the time scale. Root Mean Square Deviation (RMSD) evaluated the overall changes in protein stability due to the mutation. The backbone RMSD for all atoms from the initial structure was calculated, as it is considered the primary criterion to measure the convergence of the protein system. The backbone RMSDs were calculated for both the native and mutant models from the trajectory files. The native and mutant structures showed deviations between ~0.07 nm and ~0.28 nm, achieving equilibrium after 20 ns. A significant structural deviation was observed in the mutant proteins R74C, S66W, and R245H when compared to the native protein structure. Among all four trajectories, the native protein showed the least deviation and the least RMSD value converging at ~0.21 nm. Mutants S66W and R245H showed higher deviating patterns when compared to mutant R74C. The mutant proteins S66W and R245H exhibited a deviation range from approximately ~0.25 to ~0.27 nm, whereas mutant R74C showed convergence with an RMSD value of ~0.24 nm (Fig. 2). These variations in the deviation range between the native and mutant models explain the impact of the substituted amino acid on the protein structure and thus provide a basis for further analyses. To understand the effect of the mutants on the dynamic behavior of the residues, RMSF of the native and mutant structures was also calculated (Fig. 3). From the RMSF calculation, native residues fluctuated within the range from ~0.05–0.3 nm within the entire simulation period. We observed fluctuations that are more significant in the mutant S66W (up to ~0.7 nm), followed by the R74C and R245H mutant complexes, which exhibited fluctuations of up to ~0.45 nm. In agreement with the RMSD analysis, RMSF of all the mutants notably deviated from the native structure in the entire simulation. For further validation of the above results, native and mutant proteins were subjected to the radius of gyration (Rg) analysis to measure the level of compactness. The Rg plot (Fig. 4) showed native proteins with the smallest Rg value of ~2.13 nm. Mutant R245H exhibited an Rg value of ~2.18 nm, whereas mutants R74C and S66W had almost similar Rg patterns with a maximum deviation of approximately ~2.19 nm. The results thus predicted that all three mutants, which displayed higher deviation patterns in the RMSD analysis, also showed the highest radius of gyration values compared with the native protein, indicating a loss of compactness.

### Effects of mutations on hydrogen bonds and solvent accessible surface area

**Fig. 5:**
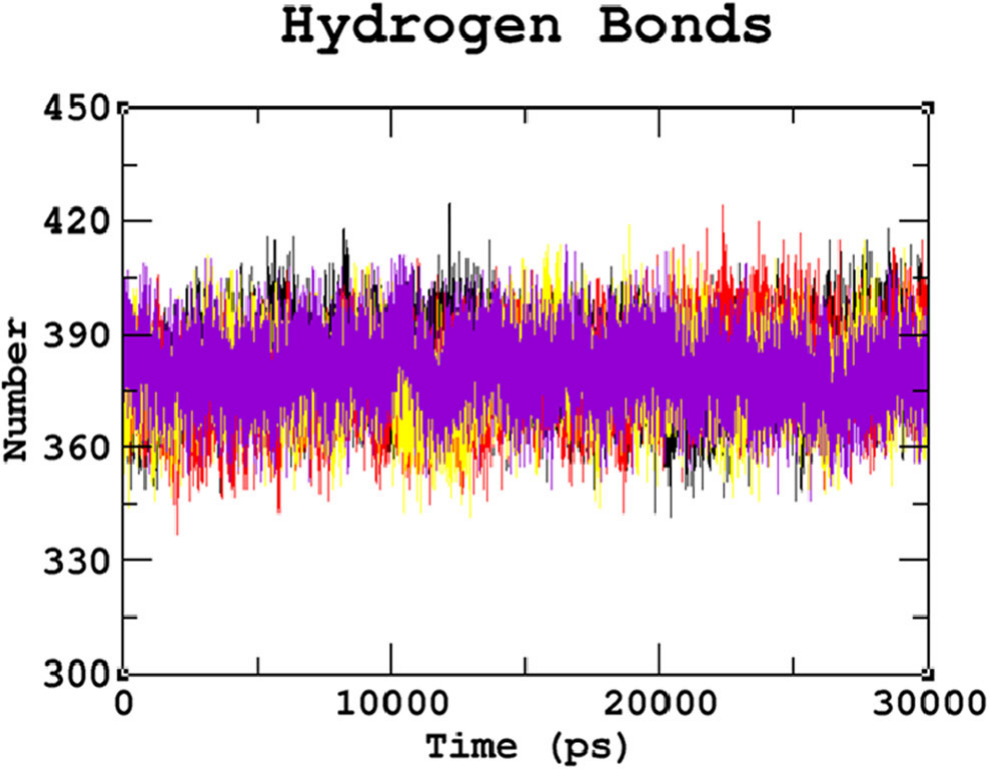
Hydrogen bond graph for the 30ns MDS of native and mutant proteins. Color scheme: (**a**) native (black), (**b**) R74C (red), (**c**) S66W (yellow), and (**d**) R245H (violet)

**Fig. 6:**
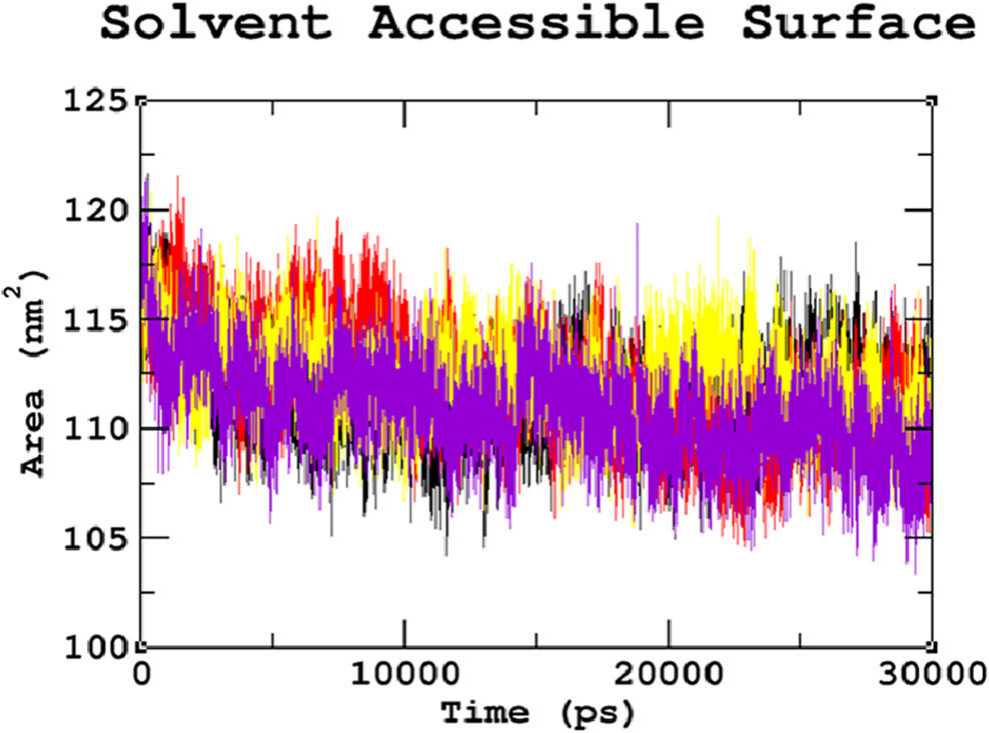
SASA graph for the 30ns MDS of native and mutant proteins. Color scheme: (**a**) native (black), (**b**) R74C (red), (**c**) S66W (yellow), and (**d**) R245H (violet)

Hydrogen bonds are one of the most critical interactions in biological processes, which help in maintaining the stability of the protein. These nsSNPs can affect the normal function of the protein by altering hydrogen bond formation (Zhang et al. 2010). The results showed a considerable difference in the number of hydrogen bonds formed between native and mutant proteins (Fig. 5). The average number of hydrogen bonds per time frame was found to be 382.530 for native, 379.147 for R74C, 378.896 for S66W, and 380.366 for R245H, respectively. Overall, it must be noted that fewer hydrogen bonds were formed in all the mutants when compared to the native protein. The reduced number of hydrogen bonds in mutant proteins might be due to the substitution of deleterious amino acids, which destroys the ability of SGSH protein to form hydrogen bonds, thus leading to its destabilization. Furthermore, the solvent-accessible surface area (SASA) was also calculated. The protein surface in contact with the surrounding solvent is referred to as the solvent accessible surface area. The solvation effect during protein folding determines the stability and rearrangement of the protein. Thus, SASA values of native and mutant proteins were calculated. The native protein had a SASA ranging from ~103 nm^2^ to ~118 nm^2^, whereas the mutant structures showed variations in the values of SASA. R74C, S66W, and R245H had SASAs ranging from ~105 nm^2^ to ~122 nm^2^, ~107 nm^2^ to ~119 nm^2^, and ~104 nm^2^ to ~122 nm^2 (^Fig. 6). These differences in SASA values in mutant protein thus indicated a potential repositioning of amino acid residues from accessible to buried regions or vice versa. The overall analysis of our study indicated that mutations R74C, S66W, and R245H had a strong influence on the structural conformation and dynamic behavior of the protein, revealing their association with the disease.

### Analysis of secondary structures and surrounding amino acid changes

**Fig. 7:**
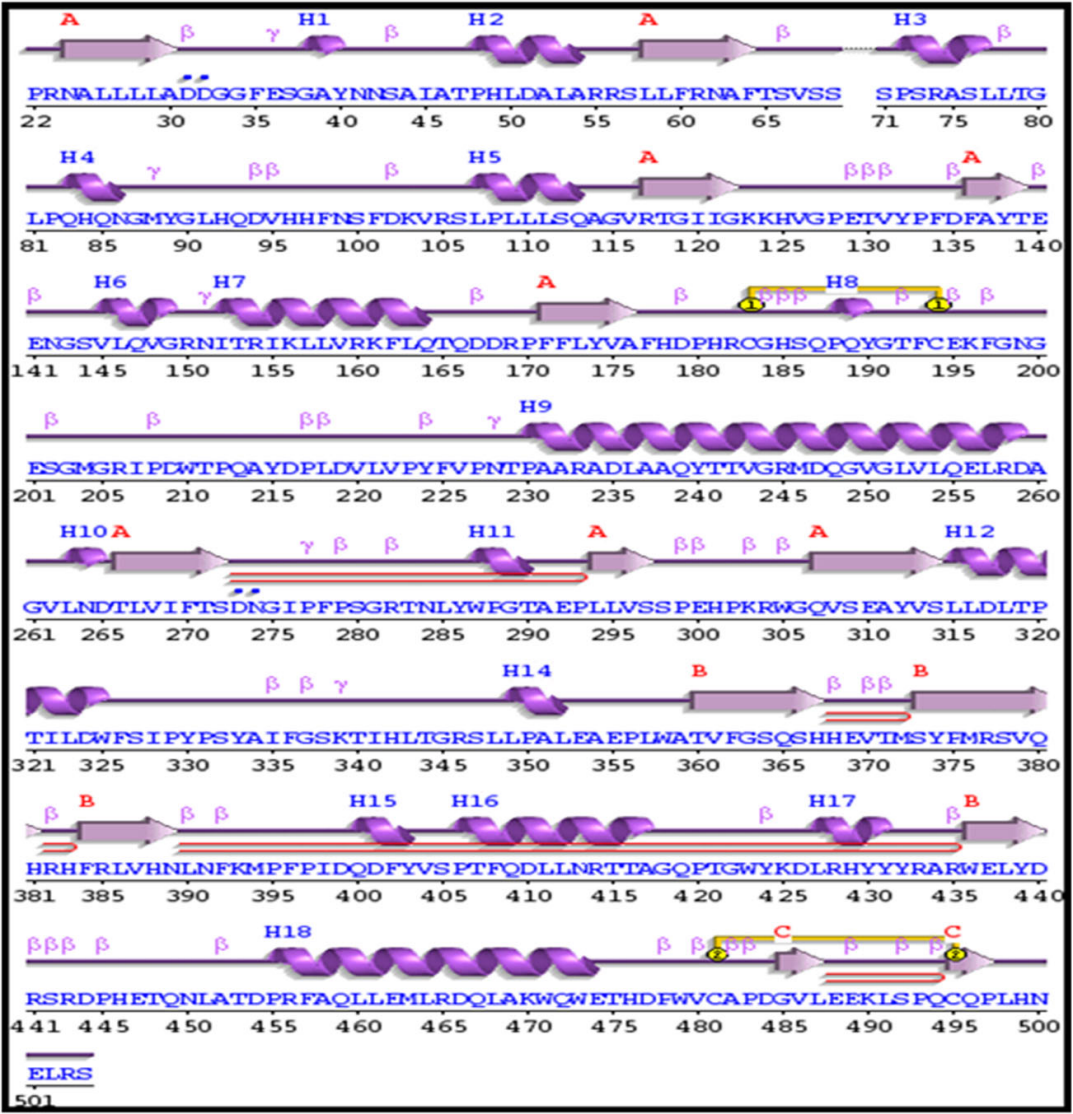
The contribution of each amino acid in SGSH protein to secondary structure elements obtained using the PDBsum database

**Fig. 8:**
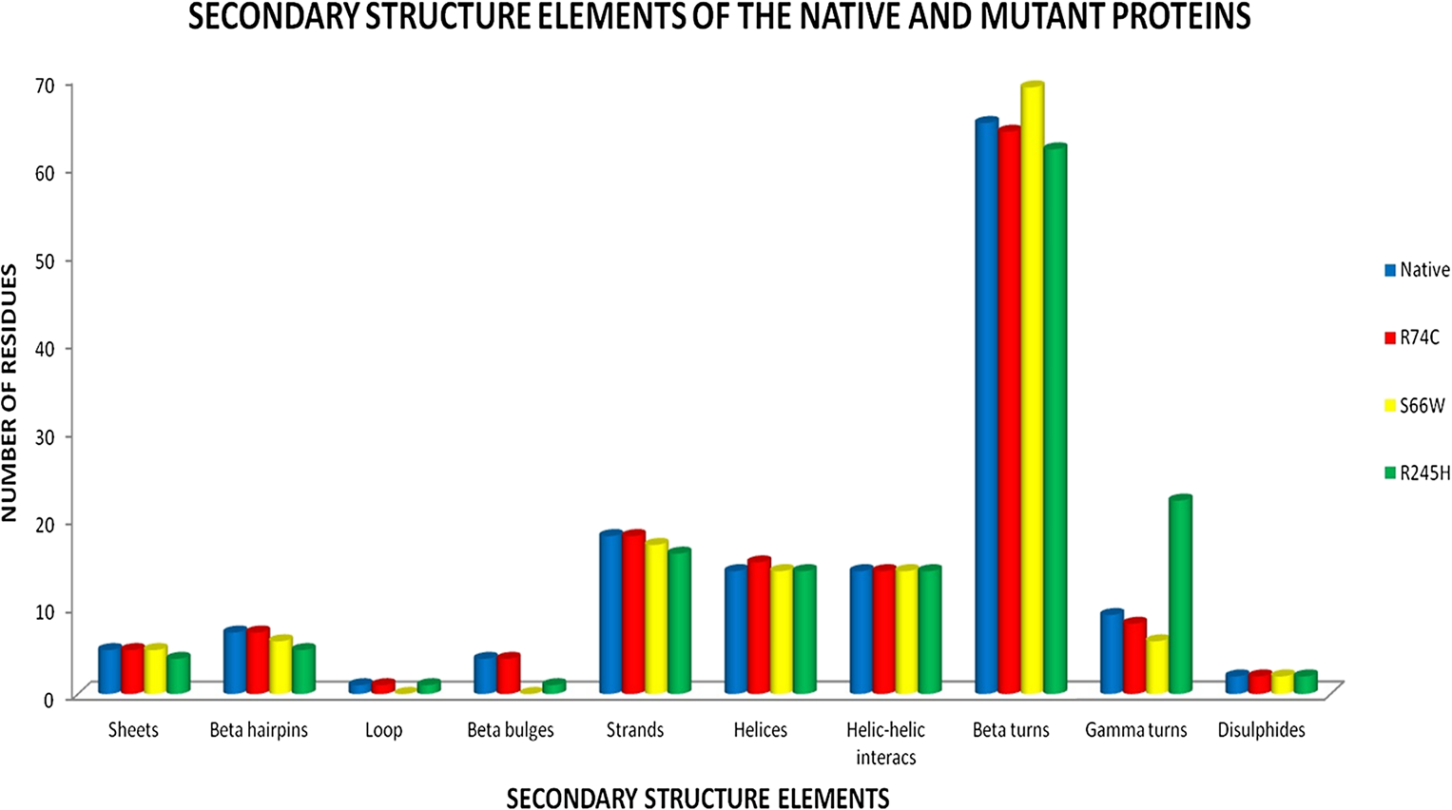
Various secondary structural elements present in the SGSH protein after the 30-ns simulation

**Fig. 9:**
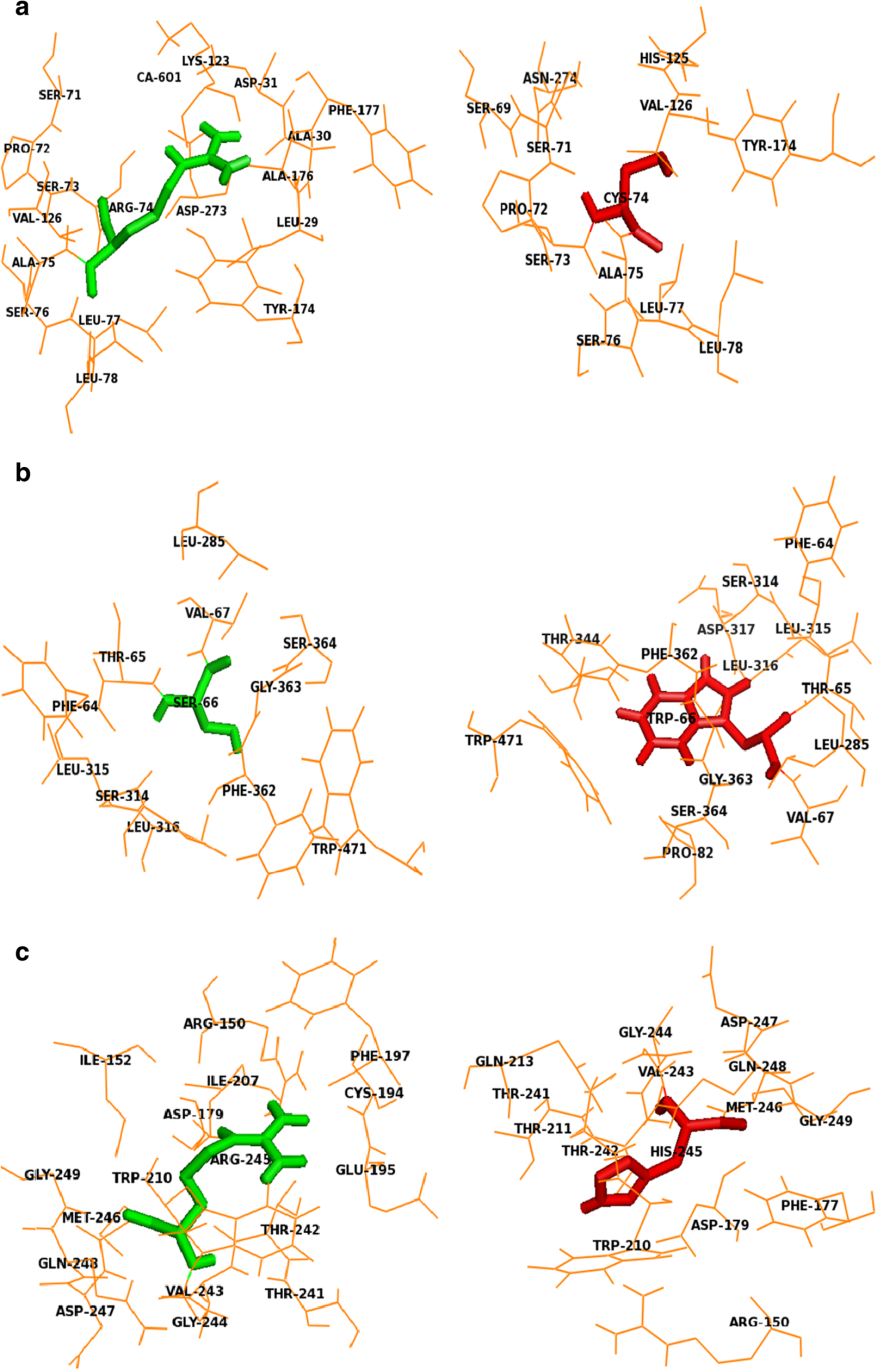
Variations in the surrounding amino acid residues in the SGSH protein by the substitution with a deleterious amino acid. (**a**) Native arginine (green) at position 74 with its surrounding amino acid residues and cysteine (red) with its surrounding amino acid residues. (**b**) Native serine (green) at position 66 with its surrounding amino acid residues and tryptophan (red) with its surrounding amino acid residues. (**c**) Native arginine (green) at position 245 with its surrounding amino acid residues and histidine (red) with its surrounding amino acid residues

Structural information plays an essential role in elucidating the molecular mechanism that leads to the disease phenotype. Since the substitution of an amino acid may induce changes at the structural level, changes in the secondary structural elements induced by mutations were analyzed using the PDBsum database. The contribution of each amino acid to the formation of secondary structure was first identified using the PDBsum database. It was observed that position S66 contributed to the formation of beta turns, whereas R74 and R245 contributed to the formation of alpha helices (Fig. 7). Figure 8 displays the changes in the number of secondary structural elements such as alpha helices, beta hairpins, beta sheets, beta bulges, strands, helix-helix interactions, gamma turns and beta turns calculated for both native and mutant structures of SGSH protein obtained at the end of the 30ns simulation. The variations were found in almost all elements of the secondary structure, except helix-helix interactions and disulfide bonds. There was a slight decrease in the number of beta-sheets in the R245H mutation. The native and mutant R74C & S66W proteins exhibited five beta sheets, whereas mutant R245H had four beta sheets. The number of beta-hairpins and strands decreased in mutants S66W and R245H when compared to the native protein and mutant R74C. A slight increase in helices was observed in R74C in comparison to the native protein and mutants S66W & R245H. The number of beta turns was 65 in the native protein, which increased to 69 in mutant S66W and decreased to 64 & 62 in mutants R74C and R245H, respectively. The native had nine gamma turns, which decreased to 8 and 6 in mutants R74C and S66W, respectively, whereas it increased to 22 in the R245H mutant. These drastic changes in secondary structural elements further confirmed that these alterations might induce an overall change in the secondary structure of the protein. Furthermore, the amino acid residue changes within 4Å surrounding the point of the mutational position at the end of the simulation were also visualized using PyMOL. Loss or gain of surrounding amino acids was observed to analyze the impact of mutations (Fig. 9A-C). Native S66 was found to interact with 11 neighboring residues (LEU316, LEU315, SER314, PHE64, THR65, VAL67, LEU285, SER364, GLY363, PHE362, and TRP471), whereas mutant S66W interacted with 14 neighboring residues (LEU316, LEU315, SER314, PHE64, THR65, VAL67, LEU285, SER364, GLY363, PHE362, TRP471, THR344, ASP317, and PRO82), showing a gain of 3 residues (THR344, ASP317, and PRO82). Native R74 interacted with 17 neighboring residues (SER71, PRO72, VAL126, ALA75, SER76, SER73, LEU77, LEU78, TYR174, ASP273, LEU29, ALA176, ALA30, PHE 177, ASP31, LYS123, and CA601). A loss of 8 residues (ASP273, LEU29, ALA176, ALA30, PHE177, ASP31, LYS123, and CA601) and gain of 3 residues (HIS125, ASN274, and SER69) were observed in mutant R74C. In native R245, there were 16 interacting residues (ILE52, ILE207, ARG150, PHE197, CYS194, GLU195, THR241, THR242, ASP179, VAL243, GLY244, ASP247, MET246, GLN248, GLY249, and TRP210), whereas in mutant R245H, a loss of 5 residues (ILE152, ILE207, PHE197, CYS194, and GLU195) and gain of 3 residues (PHE177, THR211, and GLN213) were observed. These changes in the surrounding amino acid residues further confirmed the impact of the amino acid substitutions on the structural stability of the protein.

### Principal component analysis

**Fig. 10:**
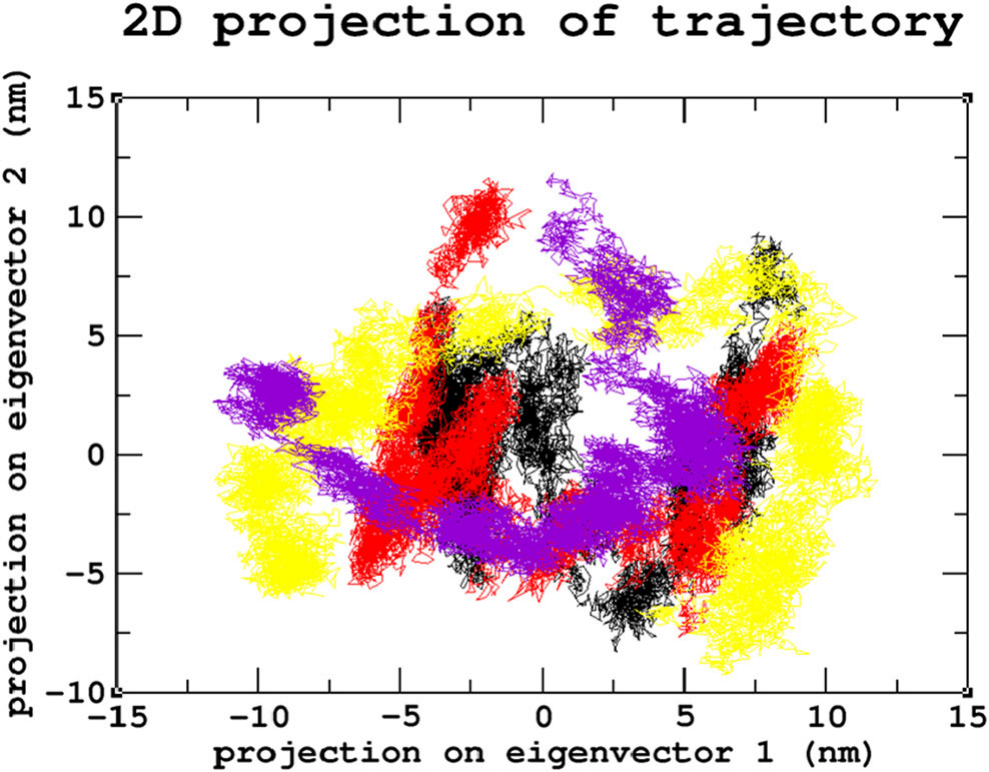
Principle component analysis graph for the 30ns MDS. Color scheme: (**a**) native (black), (**b**) R74C (red), (**c**) S66W (yellow), and (**d**) R245H (violet)

Principal Component Analysis (PCA) or Essential Dynamics (ED) was conducted to understand the variations in movements during the 30ns simulations. Initially, the covariance matrix was constructed using the eigenvalues and eigenvectors, which facilitates the PCA analysis and further represents the motional changes in the protein. These overall motions of protein atoms are associated with protein stability and aid in the function of the protein (Theobald and Wuttke 2008). PCA examines the collective motion distribution along the first two eigenvectors in the essential subspace over the entire simulation. The mutant proteins were found to cover a larger conformational space when compared to the native protein, indicating an overall increase in flexibility of the mutants (Fig. 10).

## Discussion

Prediction of the phenotypic consequences of nsSNPs using *in silico* algorithms might provide a significant understanding of the genetic differences in susceptibility to disease and response to drugs. Understanding the molecular basis of the disease at a structural level by experimental methods requires a large amount of effort and time. Since these methods have their limitations, there is a niche for *in silico* methods, which can analyze functional SNPs with greater accuracy and speed (Adzhubei et al. 2010; Calabrese et al. 2009; PS et al. 2017b). The combination of various structure and sequence-based prediction methods, which use multiple algorithms, serves as a powerful tool and provides accurate and reliable predictions in identifying mutants as deleterious or neutral. Various pathogenic prediction tools, such as PANTHER, SIFT, SNAP, PhD-SNP, and Meta-SNP and stability prediction tools, such as I-Mutant 3.0, MUpro, and SDM, were used in our study to identify the deleterious nature of the variants (Table 1). Despite variations in the input and output of these methods and limitations in making predictions, the ultimate result is the differentiation of deleterious SNPs from neutral ones. The assimilation of these techniques together increases their overall power of prediction. However, supportive evidence is necessary for validation of these prediction methods. Based on experimental studies (Esposito et al. 2000; Héron et al. 2011; Knottnerus et al. 2017; Muschol et al. 2004; Perkins et al. 1999; Sidhu et al. 2014; Trofimova et al. 2014; Weber et al. 1997), we selected three mutants R74C, S66W, and R245H for our prediction analysis. As predicted by the multiple sulfatase sequence alignment, R74 is the analogous residue in the SGSH protein. The residual activity levels of the mutant protein were found to be reduced to less than 1% of wild type SGSH protein (Yogalingam and Hopwood 2001). The replacement of a basic positively charged arginine residue with a non-polar cysteine residue would disturb the ionic interaction of the native protein. The mutant residue is smaller and more hydrophobic. This difference in size and hydrophobicity between the native and mutant protein would remove a stabilizing hydrogen bond, which is vital for hydrolysis of the sulfate ester present at the non-reducing end of the substrate. Thus, this mutation is likely to abolish the enzyme function, thus reflecting its deleterious nature. The reduced specific activity and increased susceptibility to degradation may be due to the destabilization of the active site (Perkins et al. 1999). The evolutionary stability studies and mutational resistance of protein-coding genes have demonstrated that arginine, leucine, and serine are the primary amino acids affecting protein stability in the mutants (Prosdocimi Francisco 2007). Arginine is a hydrophilic amino acid and located in the exposed region, as shown in Fig. 1. Reports suggest that proteins have evolved to place arginine residues at their surfaces to help stabilize their structures (Strub et al. 2004). Arginine is considered the most favored amino acid due to its capacity to interact in different conformations, its side chain length, and its ability to produce good hydrogen-bonding geometries (Luscombe and Thornton 2002). Thus, the substitution of arginine with cysteine could cause adverse effects on the protein conformation and significantly change the structure and function of the active site of the SGSH protein.

The amino acid residue S66 is not conserved between SGSH protein and other sulfatases. It lies near the CSPSR motif and is therefore in the coordination sphere for the cysteine residue, which is post-translationally modified in the active site of eukaryotic sulfatases (Hopwood and Ballabio 2001; Schmidt et al. 1995). Reports have shown a rapid degradation and reduced activity of the S66W mutagenized form of SGSH. The substitution of the small polar serine with the non-polar bulkier tryptophan might distort the active site, resulting in lower specific enzyme activity and stability of the protein (Weber et al. 1997). Based on sequence comparison with arylsulfatase B following superimposition, amino acid residue R245 has been hypothesized to lie near the surface of the protein on α-helix 7 of arylsulfatase B, away from the coordination sphere forming the active site. The R245H mutation will, therefore, possibly affect the stability of sulfamidase without changing the specific activity of the protein. The size difference between the native and mutant residue may alter the hydrogen bond as the native did and destabilize the local structure and packing. The difference in charge will disturb the ionic interactions of the native protein, causing a loss of interactions with other molecules and in turn leading to a possible loss of external interactions (Perkins et al. 1999; Perkins et al. 2001).

To validate the accuracy of our prediction tools, the mutants were then subjected to studies of the behavior of the protein. *In silico* analysis techniques in our study, including stability changes, pathogenic effects, and evolutionary conservation analysis, predicted that these three mutations (R74C, S66W, and R245H) had stability and functional impacts on the protein. The evolutionary analysis derives some essential features from predicting the impact of nsSNPs. The role of functional SNPs within the evolutionarily conserved regions has been validated in various studies. Deleterious mutations are more likely to correlate to protein sequences that are evolutionarily conserved due to their functional importance (Aly et al. 2006; Doniger et al. 2008; Tavtigian et al. 2008). Consequently, in our study, arginine at positions 74 and 245 and serine at position 66 were predicted to be highly conserved, functional, and exposed residues with a score of 9 based on the conservation scale of the Consurf server, illustrating the deleterious nature of mutations creating an impact on protein function (Fig. 1). The number of salt bridges formed was also compared between the native and mutant structures. Since salt bridges are dynamic and mostly exposed to the surface, they experience large thermal fluctuations and continuously break and reform. The formation of salt bridges governs the flexibility of the protein, and these salt bridge interactions are considered an essential factor in the stability of the protein (Jelesarov and Karshikoff 2009). We observed 33 salt bridges in native and mutant S66W, whereas mutants R74C and R245H had 30 and 32 salt bridges, respectively. The reduction in salt bridge formation in the mutants thus indicates the deleterious impact on protein structure and function.

Serine is a hydrophilic amino acid with hydrogen binding potential. It actively participates in hydrogen bond formation. The decrease in hydrogen bonds in mutant S66W could have been due to its substitution with a hydrophobic amino acid, tryptophan, with different physicochemical properties. Polar amino acids are commonly located in exposed regions of the protein, and any mutation in this region interferes with the functionality of the protein (Sudhakar et al. 2016). As S66 is present in the exposed region (Fig. 1), its contribution to solvent accessibility was reduced due to its substitution with tryptophan. Mutant S66W showed less solvent accessibility than the other two mutants, R74C and R245H, thus losing its contact with the surrounding solvent, as evidenced in the SASA analysis. Similarly, in the case of mutations R74C and R245H, arginine is a hydrophilic amino acid and is located in the exposed region of the protein (Fig. 1). Reports suggest that the replacement of hydrophobic residues with arginine at protein surfaces stabilizes the protein (Strub et al. 2004). Arginine interacts with the solvent and increases stability. Thus, the substitution of arginine with a hydrophobic amino acid cysteine might decrease stability and lead to a destabilization of the protein, consistent with the results obtained in the RMSD, hydrogen bond, and Rg analyses. Arginine, which has a positive charge, is larger than cysteine with a neutral charge. This difference in size and charge between the native and mutant residue might disrupt interactions with metal CA, as observed in the surrounding amino acids where the interaction with CA was lost. The difference in charge would also alter ionic interactions of the native protein, as validated by salt bridge analysis where three salt bridges were lost (Table 3). In mutant R245H, histidine is smaller than arginine. There was a decrease in the number of hydrogen bonds formed in all the mutants, as evidenced in the hydrogen bond analysis of the MDS (Fig. 4). The stability difference caused by the mutations was further studied by analyzing the changes in secondary structural elements between the native and mutant proteins using the PDBsum database. The mutational positions R74, S66, and R245 in SGSH protein were initially located. Position S66 contributed to the formation of beta turns, whereas R74 and R245 were present in the alpha-helical region of the protein (Fig. 7). The mutational positions in the secondary structure of the proteins play an essential role in identifying structural alterations in the protein (Mosaeilhy et al. 2017a; Mosaeilhy et al. 2017b; Sneha et al. 2018a; Sneha et al. 2018b; Thirumal Kumar et al. 2016; Yagawa et al. 2010; Zaki et al. 2017a). Alpha helices and beta strands are stabilized by hydrogen bonds (Schneider and Kelly 1995). Mutations that occur in alpha helix regions and beta sheets of the protein create a deleterious impact on the protein (Sneha et al. 2017a; 2017b; Mosaeilhy et al. 2017a; Mosaeilhy et al. 2017b), whereas mutations in turns or loops have minimal effects on the structural integrity of the protein (Yagawa et al. 2010). Thus, these mutations in alpha helices and beta turns could affect hydrogen bond formation and exert a deleterious impact on the protein, as validated by the hydrogen bond analysis.

Stability is a fundamental criterion that strengthens the biomolecular functions, regulation, and activity of the protein (Chen and Shen 2009). Deleterious nsSNPs can alter the normal function of a protein by changing the geometric constraints and hydrophobicity and disrupting hydrogen bonds and salt bridges (Rose and Wolfenden 1993; Shirley et al. 1992). To understand the stability and dynamic behavioral changes at an atomistic level, MDS analysis was carried out to study the behavior of the native protein and mutants R74C, S66W, R245H. Different parameters, such as RMSD, RMSF, hydrogen bond numbers, the radius of gyration, and SASA, were calculated from the simulation trajectory. Molecular stability and flexibility changes were observed based on the RMSD and RMSF analyses, respectively. The results of the SGSH protein stability analysis indicated that all the mutants (R74C, S66W, and R245H) exhibited different RMSD values when compared to the native protein. Higher deviations were observed in all mutants in comparison to the native protein. A high or reduced deviation indicates a decrease or increase in the stability of the molecule (Yun and Guy 2011). Since higher deviations led to an increase in protein rigidity, the stability analysis revealed that the mutant structures resulted in increased rigidity of the protein due to the substitution of deleterious amino acids, which was also correlated with the reduced number of hydrogen bonds in all mutants (Fig. 2a and b). Mutant S66W showed the greatest fluctuations followed by mutants R74C and R245H, thus increasing the rigidity of the protein. Thus, consistent with the RMSD analysis, the flexibility changes observed by RMSF revealed that the native protein had minimum fluctuations. As hydrogen bonds are responsible for stabilizing the structure of the protein, the determination of hydrogen bonds provides a robust and reliable indicator of the stability of the protein (Gerlt et al. 1997). Thus, the mutants showed a loss of stability by the formation of fewer hydrogen bonds than the native structure, which showed the largest number of hydrogen bonds. The reduction in a number of hydrogen bonds in the mutant structures might be due to the loss of surrounding amino acids. In the case of S66W, serine, a polar amino acid, participates in hydrogen bond formation. Serine substitution with tryptophan results in fewer hydrogen bonds, thus leading to reduced stability of the protein. Furthermore, the compactness of the protein was studied using Rg. The graph shows that the native protein had superior compactness to the mutant proteins, as evidenced by the RMSD stability analysis. The loss of surrounding amino acids in mutants could have been a reason for this loss of compactness. The SASA values were also calculated for the native and mutant structures. The observed changes in SASA values indicated the occurrence of amino acid residue repositioning from buried to accessible or accessible to buried regions. S66W had reduced solvent accessibility than mutants R74C and R245H, which indicated a potentially reduced chance of their interaction with other molecules. Thus, the SASA analysis suggested how the incorporation of deleterious amino acids introduced changes in hydrophilic and hydrophobic regions of the protein. Furthermore, based on our PCA analysis, the mutants had greater flexibility than the native protein. Greater motional changes make a protein less stable. The PCA results indicated the least stability in all mutant structures compared with the native protein, which is consistent with the results of the RMSD and hydrogen bond analyses. These motional changes indicate a loss of stability of the mutant proteins, including changes in their physicochemical properties. Therefore, the present results correlate well with experimental studies of the severity of the disease. The overall results indicated a loss of stability and functionality of the protein due to the deleterious impact of the amino acid substitutions, which might adversely affect the enzymatic activity of the protein to lead to neurodegeneration.

## Conclusion

MPS IIIA is a genetic metabolic disorder characterized by progressive neurodegeneration and behavioral problems. Understanding the relationship between genotype and phenotype based on single nucleotide polymorphisms was the most critical part of this research. Experimental methods are time consuming and laborious. Therefore, computational methods were adapted to achieve rapid and accurate predictions. In the present study, a series of *in silico *tools were used to predict three mutations (R74C, S66W, and R245H), which were prioritized based on the experimental studies. Furthermore, MDS along with distinct geometric tools, were adapted to study the influence of these mutations on the structural stability and compactness of the protein. The impact of these mutations was further explored through various computational studies to determine the stability of the protein structure. From the overall analyses, mutants R74C, S66W, and R245H were predicted to be responsible for structural differences compared with the native protein that may lead to a loss of stability and thus result in neurodegenerative disorder. Our study also emphasizes the importance of these computational approaches for the classification and interpretation of mutants that can make the process of designing personalized medicine less complicated for treatment of the disease caused by a particular mutation. Computational biology in recent years has developed the potential to speed up the drug discovery process. Identifying deleterious nsSNPs may also help in elucidating the pattern of the disease and drug response.
